# Associations of Physician Characteristics with Sex Difference in Ischemic Heart Disease Incidence among Patients Living with Type 2 Diabetes in Taiwan

**DOI:** 10.3390/healthcare9040440

**Published:** 2021-04-08

**Authors:** Yung-Hsin Lee, Ya-Hui Chang, Li-Jung Elizabeth Ku, Jin-Shang Wu, Muhammad Atoillah Isfandiari, Li-Ping Chou, Chung-Yi Li

**Affiliations:** 1 National Institute of Cancer Research, National Health Research Institutes, Tainan 704, Taiwan; justlovegod@gmail.com; 2 Department of Public Health, College of Medicine, National Cheng Kung University, Tainan 701, Taiwan; yhccat@gmail.com (Y.-H.C.); eljku@mail.ncku.edu.tw (L.-J.E.K.); 3 Department of Family Medicine, School of Medicine, College of Medicine, National Cheng Kung University, Tainan 701, Taiwan; jins@mail.ncku.edu.tw; 4 Department of Family Medicine, National Cheng Kung University Hospital, Tainan 704, Taiwan; 5 Department of Epidemiology, Faculty of Public Health, Universitas Airlangga, Surabaya 60115, Indonesia; muhammad-a-i@fkm.unair.ac.id; 6 Department of Cardiology, Tainan Sin-Lau Hospital, Tainan 701, Taiwan; m3615@ms27.hinet.net; 7 Department of Health Care Administration, Chang Jung Christian University, Tainan 711, Taiwan; 8 Department of Public Health, College of Health, China Medical University, Taichung 406, Taiwan; 9 Department of Healthcare Administration, College of Medical and Health Science, Asia University, Taichung 413, Taiwan

**Keywords:** diabetes mellitus, cardiovascular diseases, continuity of patient care, medication adherence, physician characteristics

## Abstract

(1) Background: Certain non-biological factors are suspected to explain the reduced sex difference in cardiovascular risk after diabetes. This study aimed to assess whether physician characteristics may account for such reduced sex difference. (2) Methods: Totally 10,105 type 2 diabetes patients (including 4962 men and 5143 women) were selected from Taiwan’s National Health Insurance claim data. The three-year period following the first day of clinical visit for type 2 diabetes in 2000 was set as the baseline period. The follow-up was made from the first day after baseline period to date of ischemic heart disease (IHD) incidence or censoring. Cox regression model was used to estimate hazard ratios (HRs) of IHD in relation to physician’s characteristics. (3) Results: The incidence of IHD for men and women was estimated at 17.47 and 15.96 per 1000 person-years, respectively. After controlling for socio-demographic variables and co-morbidity, male patients experienced a significantly higher HR than females for IHD (1.16, 95% Confidence Interval (CI) 1.04 to 1.29). Further adjustment for treatment adherence/continuity and physician characteristics resulted in essentially the same results. (4) Conclusions: Our study provides little support for the notation that physician characteristics may contribute to the reduced sex difference in IHD incidence in patients with type 2 diabetes.

## 1. Introduction

Compared to men, women have a much lower risk of cardiovascular disease (CVD) through the entire lifespan. However, women were found to experience a greater increased risk for cardiovascular complications than men after diabetes [1,2,3,4]. Peters et al. [1] conducted a meta-analysis examining the relationship between diabetes and incident coronary heart disease (CHD) in men and women, respectively, and found that the relative risk (RR) for incident CHD associated with diabetes was significantly elevated at 2.82 for women and 2.16 for men. The multiple-adjusted RR ratio for incident CHD was 44% greater in women with diabetes than in men with diabetes. In another meta-analysis, Peters et al. [2] noted that the RR of stroke associated with diabetes was significantly increased at 2.28 in women and 1.83 in men. Again, pooled data indicated that compared with men with diabetes, women with diabetes had a greater RR for incident stroke, with a magnitude of 27%.

Although not fully illustrated, several reasons have been proposed to explain such a “catch-up” effect by women after diabetes, including contribution of sex hormones and sex-specific risk factors [5,6,7]. Meanwhile, some non-biological reasons, including physician and patient characteristics and behaviors, have also been suspected to be associated with such sex difference in RR of CVD. Previous studies showed that women with diabetes encountered more barriers than male diabetes to have access to appropriate and cardio-protective medical care [8,9,10,11]. The UK National Diabetes Audit reported that compared to male patients, female diabetes is 15% less likely to receive medical care recommended by the treatment guidelines or to meet diabetes care targets [9].

Additionally, a previous US study noted that women with diabetes were 25% less likely to achieve target cholesterol levels than men, suspected to be due to inadequate medication for women patients [8]. Moreover, Kirkman et al. [12] conducted a large-scale study in the US and its territories and found that adherence to antidiabetic medication was slightly lower among women. The authors commented that it is very likely that physicians have been liable to treat cardiovascular disease as predominantly a ‘man’s disease,’ which is the traditional opinion, and some physicians maybe still think this way [7].

We carried out this study to test the hypothesis that physicians’ characteristics (mainly age, sex, and specialty) are associated with cardiovascular outcomes in type 2 diabetes (T2DM), which in turn accounts for the reduced sex difference in cardiovascular disease risk after diabetes.

## 2. Materials and Methods

Data were retrieved from Taiwan’s National Health Insurance (NHI) research database, a medical claim database that stores the medical records of beneficiaries and being uploaded by medical institutions to obtain reimbursement from NHI program. The NHI program universally covers the medical insurance of nearly all (>99%) Taiwanese residents [13]. The National Health Insurance Administration performs quarterly expert reviews on a random sample of medical claims to ensure their accuracy [14].

The NHI claims provide encrypted patient identification number (PIN), gender, birthday, date of ambulatory visit, date of admission and discharge (for inpatient claim), medical institutions providing health care services, *International Classification of Diseases*, *Ninth Revision*, *Clinical Modification* (ICD-9-CM) diagnostic and procedure codes, outcome at hospital discharge (recovered, died, or transferred), and co-payments charged to patients. Medical orders such as drugs prescribed and laboratory work being ordered were also included. Information on medical personnel (including physicians and other healthcare workers), including licensed date, specialty, city/township of practice, and encrypted PIN, is also available. Availability of research data was ethically approved by the Review Committee of National Health Research Institutes.

### 2.1. Research Design and Study Cohorts

This was a claim data-based retrospective cohort study. The T2DM group included all patients who sought ambulatory care for T2DM (International Classification of Disease, 9th revision, Clinical Modification ICD-9-CM: 250.×0 or 250.×2) in 2000 (*n* = 37,559). Patients who had <2 outpatient visits for T2DM or had a time period between first and last outpatient visits for T2DM <30 days (*n* = 10,411) were excluded in order to avoid potential disease miscoding [15]. Additional criteria for exclusion include: <18 years of age at cohort entry (i.e., date of first T2DM diagnosis in 2000) (*n* = 104), with a history of cardiovascular disease including coronary artery disease (ICD-9-CM: 410–414 and 428; A-code: A291, A292, A293, A299), cerebral vascular attack (ICD-9-CM: 430–436), and coronary revascularization procedures (ICD-9-CM: 36.0, 36.01, 36.02, 36.05, 36.06, 36.1, 36.10–36.19) prior to cohort entry (*n* = 8314), termination of insurance policy within a three-year period after cohort entry (*n* = 837), and with type 1 diabetes diagnosis within three years after cohort entry (*n* = 600).

**Figure 1:**
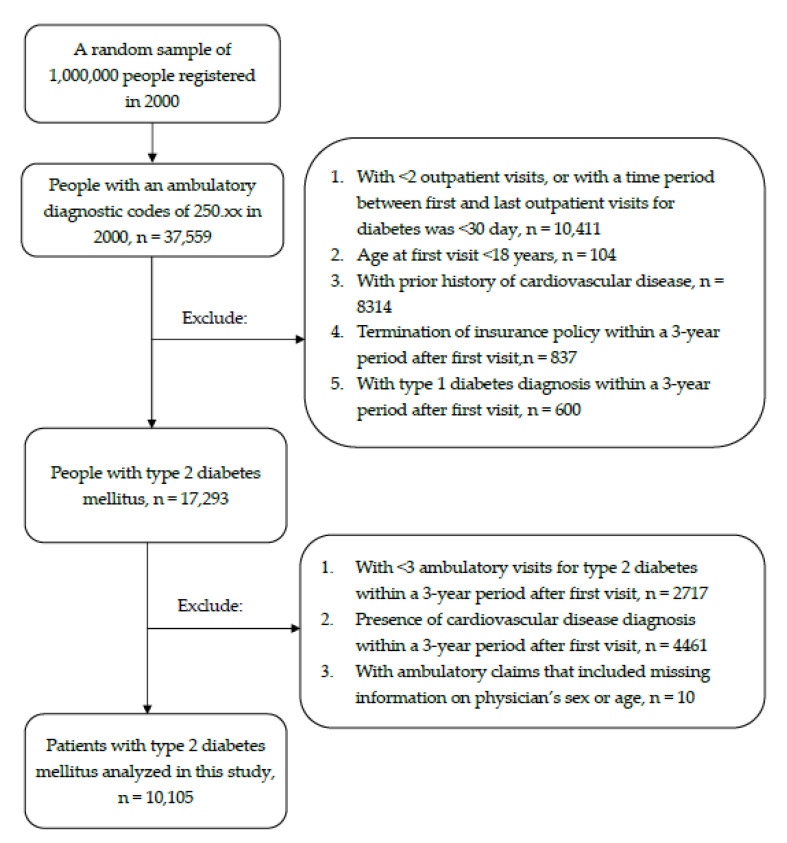
Flow chart of study subjects’ enrolment.

We set a three-year period after cohort entry as the baseline period to determine the primary physician for each T2DM patient. In doing so, we further excluded: (1) those who had <3 ambulatory visits for T2DM within baseline period (*n* = 2717); (2) those who developed cardiovascular disease within baseline period (*n* = 4461); and (3) Patients with missing information of physician’s gender and age in their medical claims (*n* = 10). The study subjects finally consisted of 10,105 patients with T2DM (4962 men and 5143 women) who can be either incident or prevalent cases at cohort entry. Figure 1 shows the flow chart of study subjects’ enrolment.

### 2.2. Physician Characteristics

The physician who provided the highest number of ambulatory care visits for specific patient within the baseline period was regarded as the primary physician for this patient. If more than one physician shared the same number of ambulatory care visits, the physician who cared the patient at the earliest time within the baseline period was selected as the primary physician. Age and specialty of the primary physician for each T2DM patient was determined on date of cohort entry. A physician identified may have cared for multiple patients among the study subjects.

### 2.3. Follow-Up and Outcome Ascertainment

Follow-up started from the first day (i.e., index date) after the baseline period. We linked the study subjects, using the unique PIN, to ambulatory and inpatient claims (2000–2013) in an attempt to identify the possible incidence of primary or secondary diagnoses of ischemic heart disease (IHD, [ICD-9-CM: 410–414]).

### 2.4. Covariates

Socio-demographic variables, co-morbidity, medication adherence, and continuity of care were the major categories of covariates considered in this study. Information of monthly income and residential area indicated by city district/township was retrieved from the 2000 Registry of Beneficiaries of NHI program. We categorized all residential areas into four geographic locations, i.e., northern, central, southern, and eastern. The classification scheme used to determine level of urbanization for each residential rea was proposed by Liu et al. [16]. Consideration of urbanization level and neighborhood socioeconomic status was made to account for differential diagnostic techniques in different areas; and for varying health care accessibility [17].

Selected co-morbidity included hypertension (ICD-9-CM: 401–405), obesity (ICD-9-CM: 278.0 or 278.1), and Diabetes Complication Severity Index (DCSI). DCSI was calculated from diabetic complications, including retinopathy, nephropathy, neuropathy, cerebrovascular, cardiovascular, peripheral vascular, and metabolic disorders [18,19].

The medication possession ratio (MPR) was measured by level of adherence to anti-diabetic drugs on the basis of the refill pattern. The number of prescription days was estimated from the quantity of drugs from Pharmacy claims in the National Health Insurance Research Database (NHIRD); and MPR was computed by dividing the total number of prescription days the patient received by 1095 days.

The continuity of care [20] score was calculated based on the outpatient services only. Considering the variation and very high numbers of physician visits in Taiwan, we chose the continuity of care index (COCI) as our covariate because the COCI is minimally sensitive to the number of physician visits by patients. The COCI value ranges between 0 and 1, with a higher value indicating better continuity of care.

In order to improve the quality-of-care and decrease medical expenditures, Taiwan’s NHI program implemented the diabetes pay-for-performance (P4P) program in 2001, which provides participant interventions and services including medical history assessment, physical examination, bio-physiological tests, creation of management plan, and DM self-management education [18,21]. While the enrolment of diabetes patients in the P4P program is largely a physician’s decision, the NHI program offers higher payment if diabetes patients can achieve relatively higher scores of selected performance indicators, including the percentages of recruiting new patients (≥30%), well-controlled cases (HbA_1C_ < 7%), poor-controlled cases (HbA_1C_ > 9.5%), and low-density lipoprotein (LDL) > 130 mg/dL in a year. This additional incentive drives physicians to pursue better quality of care [22].

### 2.5. Statistical Analysis

A comparison of patient and physician characteristics, presented as number/percentage and mean/standard deviation for categorical and continuous variables, respectively, between T2DM patients and controls was firstly made. Then, overall and covariate stratified incidence rates, under the Poisson assumption, of IHD were calculated. We performed sequential multivariate Cox proportional hazard models to estimate the covariate adjusted HR of IHD in association with the patient’s gender. Socio-demographic variables, medication adherence, continuity of care, and physician characteristics were sequentially included in the regression model to assess how these covariates might have posed influence on the association of patient’s gender with hazard of IHD.

Using a multiplicative model, we further performed tests for interactive effect of physician characteristics with patient’s gender on IHD hazard. We also compared medication adherence, continuity of care, and P4P enrolment between male and female patients according to the physician’s gender. We examined the assumption of “*proportionality*” by plots of *log(−log(survival function))* vs. *log(time)*, and found no violation for the Cox proportional hazard models conducted in our analyses. The data were analyzed using SAS (version 9.4; SAS Institute, Cary, NC). A *p* value of 0.05 or less was considered statistically significant.

## 3. Results

Male (*n* = 4962, 49.1%) and female (*n* = 5143, 50.9%) patients with T2DM were similar in most of the socio-demographic characteristics, except urbanization status of residential city district/township in which more men than women lived in urban areas. In spite of little difference in DCSI score, men tended to have lower prevalence of hypertension and obesity than women, which is likely due to a higher percentage of incident of T2DM in men than in women (25.96% vs. 22.89%). While women apparently had a better MPR and a higher percentage of enrolment in P4P than men, both genders were similar in level of COCI. With respect to physician characteristics, the primary physicians of male patients were slightly older than those of female patients. On the other hand, gender and specialty of the physicians for both patient groups are essentially the same (Table 1).

Table 2 shows the crude and covariate adjusted HR of IHD in association of male gender of T2DM patients. Because the information of IHD was retrieved from the reimbursement claim data, all the IHD analyzed in our study can be considered non-fatal. Over the study period, male patients had a higher incidence rate of IHD than their female counters (17.47 vs. 15.96 per 1000 person-years). After adjustment for socio-demographic variables and co-morbidity, male patients were associated with a significantly elevated hazard of IHD (HR 1.16, 95% CI = 1.04–1.29) (Model 2). Additional adjustment for MPR, COCI, and enrolment in P4P resulted in essentially the same HR at 1.16 (95% CI = 1.04–1.30) (Model 3), which also remained unchanged after further inclusion of physician characteristics in the regression model (Model 4). In addition to male gender, the full model (i.e., model 4) also showed that older age, higher DCSI score, hypertension, non-incident cases, and lower level (<80%) of COCI were all associated with significantly higher HRs of IHD.

Table 3 presents the HRs of IHD in association with male gender of T2DM patients according to physician characteristics. Among patients whose primary physicians were male, male patients had significantly higher HR of IHD than female patients (HR 1.19, 95% CI = 1.06–1.34). The corresponding figure for patients primarily cared by female physicians was 0.95 (95% CI = 0.64–1.40). In the stratified analysis by physician’s age, the significant association of patient’s gender with IHD was only observed among patients with older (≥55 years) physicians (HR 1.49, 95% CI = 1.01–2.18). Despite the above findings, the statistical test for interaction of patient’s gender with physician’s gender (*p* value = 0.44) and age (*p* value = 0.27), respectively, was not significant statistically. The analysis stratified by a physician’s specialty also showed no significantly elevated HR for male patients regardless of the physician’s specialty (*p* value = 0.16)

## 4. Discussion

To the best of our knowledge, this is the first study that assessed the roles of non-biological factors that might have accounted for the ‘catching-up’ effect in women after diabetes. Our data showed a significantly higher (16%) risk of IHD in male T2DM than their female counterparts, which was essentially smaller than the increased risk reported from the general population of Taiwan, in a range of 21% [23] to 170% [24]. In addition, the study of the Asia Pacific Cohort Studies Collaboration, including Taiwanese patients, showed that the hazard ratios were similar in men (2.03; 95% CI 1.60–2.59) and women (2.54; 95% CI 1.84–3.49) (*p* for interaction = 0.27) for death from CHD. Additionally, the hazard ratios of cerebrovascular disease death were almost exactly the same in women (2.00; 95% CI 1.37–2.92) and men (2.04; 95% CI 1.46–2.84) [25]. The addition of medication adherence and continuity of care as well as physician characteristics to the regression model resulted in little influence on the relative risk estimate. Our study, thus, suggested little influence of physician characteristics in explaining the reduced sex difference in IHD among patients with T2DM. An earlier Taiwanese study by Chen and Li [24] reported a relative risk of IHD of 1.22 in favor of women in non-diabetes population, which reduced to 1.10 in type 2 diabetes individuals. If such reduced sex difference after diabetes can be attributable to healthcare provider characteristics, we hypothesized that the HR of IHD would increase after adjustment (standardized) for physicians’ characteristics, namely, age, sex, and specialty in this study.

Among the potential non-biological causes for the reduced sex difference in cardiovascular disease risk after diabetes, the accessibility explanation is frequently mentioned. It showed that women have received poorer cardiovascular care than men when they develop diabetes because lower accessibility to diabetes care was more common in female than in male diabetes [7]. Under the context of Taiwan’s universal health care, it is believed that the sex difference in barriers to health care have largely been removed. A recent study that examined the urban–rural difference in guideline-recommended diabetes care between 2000 and 2010 in Taiwan reported a small urban–rural gap in receiving essential examinations/tests for diabetes care [26]. In fact, our data also demonstrated that the percentage of P4P enrolment was higher in female patients (12.67–15.96%) than in males (9.83–14.84%) (Table 4).

The behavior of both patients and physicians is another possible explanation for the reduced sex difference in CVD outcome among T2DM patients. The study by Dupre et al. reported that women with cardiovascular disease tended to have lower health literacy than men with the same condition [27]. Despite that it seems unlikely that women will, in general, be less aware of the adverse consequences of unhealthy risk behaviors, relative to men [28], data from a very large study in the USA and its territories found adherence to anti-diabetic medication to be slightly lower among women [12]. Shrestha et al. conducted a cross-sectional survey of 100 men and 100 women with T2DM in western Nepal and found that women had low self-efficacy with respect to their diabetes care (35%) in comparison to men (65%). Compared to women, men were also associated with a much lower risk (by 50%) of bad dietary practices [29]. Additionally, an important contributor to sex differences in cardiovascular diseases could be related to differences in availability, reliability, and efficiency of diagnostic methods, as recently discussed in an expert opinion paper by Madonna et al. [30]. Despite the above argument, our study in fact revealed better adherence to medication and continuity of care among women patients (Table 4). Nonetheless, the slightly better medication adherence and continuity of care in female gender did not explain the apparent sex difference in IHD observed in our study.

Apart from patient characteristics, a number of studies were conducted to investigate physician characteristics in relation to health outcomes. Tsugawa et al. studied 736,537 admissions managed by 18,854 hospitalist physicians [31]. After adjustment for the characteristics of patients and within the same hospital, patients treated by older physicians had higher mortality than patients cared for by younger physicians, except those physicians treating high volumes of patients. In another study, Tsugawa et al. included an even greater number of admissions (*n* = 1,583,028) to examine the 30-day mortality in association with physician gender [32]. It showed that elderly hospitalized patients treated by female internists have lower mortality and readmissions compared with those cared for by male internists. Moreover, a local Taiwanese study reported a reduced risk of acute hyperglycemic events in T2DM cared by endocrinology specialty, as compared to other internal medicine specialty and family medicine physician [33].

It is possible that some physicians may be liable to treat CVD as predominantly a ‘man’s disease,’ and thus, have overlooked the cardiovascular risk of female T2DM. We further compared the prevalence of MPR, COCI, and P4P according to patients’ and physicians’ gender. Table 4 shows that the prevalence of MPR and P4P enrolment, but not COCI, differed significantly among the four patient groups. It is noted that male patients cared for by male physicians demonstrated the poorest MPR and P4P enrolment. These findings suggest a potential influence of physicians’ gender on sex-specific diabetes care in Taiwan. Nonetheless, our analytical results did not disclose a meaningful influence of physicians’ age, sex, and specialty on the reduced relative risk of IHD in relation to patients’ gender.

Our study might have several methodological limitations. First, the information of diagnostic codes for ascertaining T2DM is subject to error, at least to some extent. In fact, an earlier local study indicated that the accuracy of a single diabetes diagnosis in the NHI claim data was only satisfactory at 74.6% [34]. In this study, we used at least two diagnoses of T2DM at outpatient settings, with the first and the last visits at least 30 days apart. This way of doing may help reduce the likelihood of disease misclassification. Second, we were unable to completely account for the known risk factors (e.g., adverse lifestyle and body mass index) for cardiovascular disease in the analysis, which might have resulted in residual confounding. Third, the majority of our study sample was prevalent rather than incident cases of diabetes, and the prevalence of prevalent cases was higher in women than in men (77.11% vs. 74.04%). As the length of diabetes is an independent risk factor for IHD, we managed to account for this potential confounding by adjusting DCSI in the regression model, as indicated in the previous studies that show that DCSI has a favorable correlation with diabetes duration [18,19]. Fourth, we did not consider medication in our analysis, which might be prescribed differentially to male and female patients. We analyzed the distributions of certain common anti-glucose drugs, including sulfonylureas, meglitinides, thiazolidinediones, α-glucosidase inhibitors, insulin, and metformin, and noted no significant differences in these medications in patient groups characterized by the gender of physicians and patients. Nonetheless, we do not have the information of other drugs that could also be related to IHD, such as aspirin and ACE inhibitors. Moreover, there is laboratory data such as glucose level and HbA1c, which also impede the assessment of potential influence of laboratory data on our study results.

Apart from the above potential sources of biases, the study was based on mainly ethnic Chinese people under the context of universal insurance coverage with a single payer, which may have largely standardized the treatment behaviors of Taiwanese physicians and contributed to little sex differences noted in Taiwan patients’ medication adherence, continuity of care of care, and physicians’ behavior. There is evidence of a gender bias in the management of type 2 diabetes, suggesting undertreatment of type 2 diabetes and other cardiovascular disease risk factors in women compared with men [35]. Women with type 2 diabetes might have more advanced atherosclerosis than men at the same stage of disease; some clinicians might not be aware that type 2 diabetes presents a greater risk for severe consequences of cardio-vascular disease in women than in men [36]. There is also a sex difference in the response to antidiabetic drugs. A stratified analysis revealed that lean men with type 2 diabetes show greater glycemic reduction with sulfonylureas (which increase insulin secretion) than lean diabetes women, whereas women with type 2 diabetes and obesity show greater glycemic reduction with thiazolidinediones (which increase insulin sensitivity) than diabetes men with obesity [37]. The potential sex difference in diabetes care and treatment response might be further overlooked in areas without universal health care coverages. Whether our findings can still be obtained elsewhere should be further investigated in other contexts.

## 5. Conclusions

In conclusion, our cohort study found little association between physician characteristics and medication adherence as well as continuity of care among patients with T2DM in Taiwan. Additionally, as physician characteristics were not profoundly associated with risk of IHD, and did not alter the relative risk estimate of IHD in relation to male gender of T2DM patients, our study suggests physician characteristics had little influence in explaining the reduced sex difference in IHD among patients with T2DM.

## Figures and Tables

**Table 1:** Characteristics of type 2 diabetes patients and their doctors.

Characteristics	Patients with Type 2 Diabetes	
	*N* = 10,105	
	Male	Female
	*n* = 4962	*n* = 5143
*Patient characteristics* *		
Age (years)		
18–44	586 (11.81)	393 (7.64)
45–64	2778 (55.99)	2704 (52.58)
≥65	1598 (32.20)	2046 (39.78)
Mean ± SD	58.97 ± 11.82	61.44 ± 11.29
Monthly income (NTD)		
Dependent	812 (16.36)	1885 (36.65)
<15,840	776 (15.64)	352 (6.84)
15,840–28,800	2304 (46.43)	2692 (52.34)
>28,800	1070 (21.56)	214 (4.16)
Geographic area		
Northern	2109 (42.50)	2239 (43.53)
Central	1102 (22.21)	1199 (23.31)
Southern	1588 (32.00)	1544 (30.02)
Eastern	163 (3.28)	161 (3.13)
Urbanization status		
Urban area	2147 (43.27)	2128 (41.38)
Satellite area	1329 (26.78)	1353 (26.31)
Rural area	1486 (29.95)	1662 (32.30)
DCSI		
0	2242 (45.18)	2068 (40.21)
1	1431 (28.84)	1575 (30.63)
2	755 (15.22)	847 (16.47)
3	33 (6.67)	428 (8.32)
≥4	203 (4.09)	225 (4.37)
Median	1	1
Hypertension		
Yes	2399 (48.35)	2788 (54.21)
No	2563 (51.65)	2355 (45.79)
Obesity		
Yes	40 (0.81)	72 (1.40)
No	4922 (99.19)	5071 (98.60)
Incident case at baseline		
Yes	1288 (25.96)	1177 (22.89)
No	3674 (74.04)	3966 (77.11)
MPR		
<80%	2756 (55.54)	2555 (49.68)
≥80%	2206 (44.46)	2588 (50.32)
Mean ± SD	68.17 ± 29.32	72.23 ± 27.91
COCI		
<80%	3529 (71.12)	3677 (71.50)
≥80%	1433 (28.88)	1466 (28.50)
Mean ± SD	0.56 ± 0.30	0.57 ± 0.29
Enrolled in P4P		
Yes	511 (10.30)	668 (12.99)
No	4451 (89.70)	4475 (87.01)
*Physician characteristics* ^†^		
Sex		
Male	4497 (90.63)	4648 (90.38)
Female	465 (9.37)	495 (9.62)
Age (years)		
<35	202 (4.07)	191 (3.71)
35–44	2400 (48.37)	2558 (49.74)
45–54	1807 (36.42)	1917 (37.27)
≥55	553 (11.14)	477 (9.27)
Mean ± SD	45.72 ± 7.93	45.40 ± 7.63
Specialty		
Endocrinology	1365 (27.51)	1468 (28.54)
Cardiology	134 (2.70)	162 (3.15)
Family medicine	809 (16.30)	809 (15.73)
Internal medicine	1248 (25.15)	1173 (22.81)
Others	1406 (28.34)	1531 (29.77)

* Inconsistency between total sample size and stratum-specific sample size summed for some patient characteristics was due to missing information. ^†^ One physician could be the primary doctor for multiple patients. SD = standard deviation; NTD = New Taiwan Dollar (1 USD ≅ 30NTD); DCSI = Diabetes Complications Severity Index; MPR = Medication Possession Ratio; COCI = continuity of care index; P4P = pay-for-performance.

**Table 2:** Overall and stratified incidence densities and hazard ratios of ischemic heart disease in association with sex and selected covariates among patients with type 2 diabetes.

	PY	No. of IHD	ID (1000 PY)	Hazard Ratio (95% CI) *			
				Model 1	Model 2	Model 3	Model 4
Overall	87,435.57	1459	19.69				
*Patient characteristics*							
Sex							
Male	42,135.65	736	17.47	1.09 (0.98–1.21)	1.16 (1.04–1.29)	1.16 (1.04–1.30)	1.16 (1.04–1.30)
Female (ref.)	45,299.92	723	15.96	1	1	1	1
Age (years)							
18–44 (ref.)	9670.88	80	8.27		1	1	1
45–64	49,778.91	856	17.2		1.93 (1.53–2.43)	1.93 (1.53–2.43)	1.92 (1.53–2.41)
≥65	27,985.79	523	18.69		1.90 (1.49–2.43)	1.90 (1.49–2.43)	1.89 (1.48–2.41)
Monthly income (NTD)							
Dependent	22,544.29	404	17.92		1.08 (0.89–1.31)	1.08 (0.89–1.31)	1.07 (0.89–1.30)
<15,840	8900.5	174	19.55		1.12 (0.90–1.39)	1.11 (0.89–1.34)	1.10 (0.89–1.36)
15,840–28,800	43,871.07	692	15.77		1.00 (0.85–1.19)	1.00 (0.85–1.19)	1.00 (0.84–1.19)
>28,800 (ref.)	12,119.72	189	15.59		1	1	1
Geographic area							
Northern (ref.)	38,142.28	638	16.73		1	1	1
Central	19,838.65	317	15.98		0.97 (0.83–1.15)	0.98 (0.83–1.15)	0.96 (0.81–1.15)
Southern	26,722.17	460	17.21		1.03 (0.90–1.18)	1.02 (0.89–1.17)	1.01 (0.88–1.17)
Eastern	2732.48	44	16.1		0.92 (0.66–1.29)	0.91 (0.66–1.28)	0.90 (0.65–1.25)
Urbanization status							
Urban area (ref.)	37,732.33	628	16.64		1	1	1
Satellite area	23,028.33	390	16.94		1.05 (0.91–1.20)	1.04 (0.90–1.20)	1.04 (0.91–1.19)
Rural area	26,674.92	441	16.53		1.06 (0.89–1.25)	1.05 (0.89–1.25)	1.05 (0.89–1.23)
DCSI							
0 (ref.)	38,559.17	552	14.32		1	1	1
1	26,473.63	438	16.54		1.13 (0.99–1.28)	1.13 (0.98–1.26)	1.13 (0.99–1.28)
2	13,308.01	251	18.86		1.25 (1.08–1.45)	1.23 (1.06–1.43)	1.24 (1.06–1.44)
3	6141.91	131	21.33		1.38 (1.14–1.68)	1.35 (1.11–1.64)	1.38 (1.13–1.67)
≥4	2952.85	87	29.46		1.85 (1.48–2.33)	1.78 (1.42–2.24)	1.81 (1.43–2.30)
Hypertension							
Yes	43,128.47	852	19.75		1.33 (1.20–1.48)	1.32 (1.18–1.47)	1.31 (1.17–1.46)
No (ref.)	44,307.1	607	13.7		1	1	1
Obesity							
Yes	1071.17	15	14		1.02 (0.61–1.69)	1.02 (0.61–1.69)	1.05 (0.65–1.68)
No (ref.)	86,364.41	1444	16.72		1	1	1
Incident case at baseline							
Yes (ref.)	21,944.54	309	14.08		1	1	1
No	65,491.03	1150	17.56		1.16 (1.02–1.32)	1.16 (1.02–1.32)	1.17 (1.02–1.33)
MPR							
<80% (ref.)	45,806.56	735	16.05			1	1
≥80%	41,629.01	724	17.39			0.98 (0.88–1.09)	0.97 (0.87–1.08)
COCI							
<80% (ref.)	61,355.49	1095	17.85			1	1
≥80%	26,080.09	364	13.96			1.19 (1.05–1.34)	1.19 (1.06–1.33)
Enrolled in P4P							
Yes (ref.)	10,703.09	175	16.35			1	1
No	76,732.48	1284	16.73			1.07 (0.91–1.26)	1.05 (0.90–1.24)
*Physician characteristics*							
Sex							
Male	79,022.65	1329	16.82				1.06 (0.87–1.29)
Female (ref.)	8412.92	130	15.45				1
Age (years)							
<35	3357.26	59	17.57				0.97 (0.69–1.38)
35–44	43,083.1	701	16.27				0.95 (0.79–1.15)
45–54	32,169.61	547	17				1.00 (0.82–1.22)
≥55 (ref.)	8825.61	152	17.22				1
Specialty							
Endocrinology (ref.)	25,286.83	399	15.78				1
Cardiology	2495.97	52	20.83				1.27 (0.94–1.71)
Family medicine	14,269.32	220	15.42				0.99 (0.83–1.17)
Internal medicine	20,702.55	368	17.78				1.10 (0.94–1.28)
Others	24,680.9	420	17.02				1.09 (0.94–1.26)

* Estimated from Cox proportional hazard model, which included all patient and physician characteristics, shown in Table 2 as the independent variables. PY = person-years; IHD = ischemic heart disease; ID = incidence density; HR = hazard ratio; CI = confidence interval; DCSI = Diabetes Complications Severity Index; MPR = Medication Possession Ratio; COCI = continuity of care index; P4P = pay-for-performance.

**Table 3:** Associations of sex with hazard ratios of ischemic heart disease according to physician characteristics.

Characteristics	Female (Ref.)				Male			*p* for Interaction
	PY	No. of IHD	ID (1000 PY)	PY	No. of IHD	ID (1000 PY)	Adjusted HR * (95% CI)	
Sex								
Male	40,913.63	654	15.98	38,109.02	675	17.71	1.19 (1.06–1.34)	0.438
Female	4386.29	69	15.73	4026.63	61	15.15	0.95 (0.64–1.40)	
Age (years)								
<35	1708.22	23	13.46	1649.04	36	21.83	1.74 (0.93–3.28)	0.266
35–44	22,506.09	360	16	20,577.02	341	16.57	1.08 (0.92–1.27)	
45–54	16,934.63	278	16.42	15,234.98	269	17.66	1.19 (0.98–1.43)	
≥55	4150.99	62	14.94	4674.62	90	19.25	1.49 (1.01–2.18)	
Specialty								
Endocrinology	13,234.52	218	16.47	12,052.31	181	15.02	1.03 (0.83–1.29)	0.161
Cardiology	1359.06	31	22.81	1136.91	21	18.47	0.73 (0.41–1.30)	
Family medicine	7222.4	102	14.12	7046.92	118	16.74	1.29 (0.97–1.73)	
Internal medicine	10,149.67	162	15.96	10,410.72	206	19.79	1.23 (0.98–1.56)	
Others	13,206.93	210	15.9	11,473.98	210	18.3	1.21 (0.99–1.49)	

* Estimated from Cox proportional hazard model, which included all patient and physician characteristics, shown in Table 2 as the independent variables. PY = person-years; IHD = ischemic heart disease; ID = incidence density; HR = hazard ratio; CI = confidence interval.

**Table 4:** Comparisons of MPR, COCI, and enrolment in P4P between patients stratified by sexes of patient and physician.

Physician’s Characteristics	Male Physician		Female Physician		*p*
	Male Patient *n* = 4497	Female Patient *n* = 4648	Male Patient *n* = 465	Female Patient *n* = 495	
Age, Mean ± SD	59.02 ± 11.81	61.54 ± 11.25	58.54 ± 11.96	60.48 ± 11.58	<0.001
MPR					<0.001
Mean ± SD	0.68 ± 0.29	0.72 ± 0.28	0.70 ± 0.29	0.72 ± 0.29	<0.001
≥80%, *n* (%)	1969 (43.78)	2343 (50.41)	237 (50.97)	245 (49.49)	<0.001
COCI					
Mean ± SD	0.56 ± 0.30	0.57 ± 0.29	0.55 ± 0.30	0.56 ± 0.28	0.252
≥80%, *n* (%)	1296 (28.82)	1326 (28.53)	137 (29.46)	140 (28.28)	0.271
Enrolled in P4P					
*n* (%)	442 (9.83)	589 (12.67)	69 (14.84)	79 (15.96)	<0.001

MPR = Medication Possession Ratio; COCI = continuity of care index; P4P = pay-for-performance.

## Data Availability

The datasets analyzed during the current study are not publicly available because of information governance restrictions.
